# Objective Structured Clinical Examination for pediatric medication administration

**DOI:** 10.15649/cuidarte.4659

**Published:** 2025-12-17

**Authors:** Mery Luz Valderrama-Sanabria, Gisella Bonilla-Santos, Daniela Muñoz-Duitama, Natalia Sofia Tellez-Avila

**Affiliations:** 1 Nurse, Professor, Universidad de los Llanos, Villavicencio, Colombia E-mail: mvalderrama@unillanos.edu.co Universidad de los Llanos Villavicencio Colombia mvalderrama@unillanos.edu.co; 2 Psychologist, Universidad Surcolombiana. Neiva, Colombia. E-mail: gisella.bonilla@usco.edu.co Universidad Surcolombiana Neiva Colombia gisella.bonilla@usco.edu.co; 3 Nurse, Universidad de los Llanos, Villavicencio, Colombia. E-mail: daniela.munoz@unillanos.edu.co Universidad de los Llanos Villavicencio Colombia daniela.munoz@unillanos.edu.co; 4 Nurse, Universidad de los Llanos, Villavicencio, Colombia. E-mail: natalia.tellez@unillanos.edu.co Universidad de los Llanos Villavicencio Colombia natalia.tellez@unillanos.edu.co

**Keywords:** Nursing, Reproducibility of Results, Drug Dosage Calculations, Pediatrics, Enfermería, Reproducibilidad de los Resultados, Cálculo de Dosificación de Drogas, Pediatría, Enfermagem, Reprodutibilidade dos Testes, Cálculos da Dosagem de Medicamento, Pediatria

## Abstract

**Introduction:**

The systematic evaluation of clinical competencies of nursing professionals has been a subject of interest among educators, professionals, and other experts in this field, which enables the tracking of the graduation profile, career trajectory, and achievement of goals throughout the professional career. Faculty must prioritize efficiency in the performance of future nursing professionals by using instruments such as clinical simulation for practice development.

**Objective:**

To determine the construct validity of the objective structured clinical examination (OSCE) for assessing competence in pediatric medication administration.

**Materials and Methods:**

An observational, psychometric, and prospective study was conducted to determine the validity and reliability of the instrument "Objective Structured Clinical Examination for pediatric medication administration."

**Results:**

The final instrument consisted of 15 items. Bartlett's test of sphericity was significant (χ2 =145.887, p < 0.001), and the Kaiser-Meyer-Olkin (KMO) measure of sampling adequacy was acceptable (0.703). Cronbach’s alpha for the total instrument was 0.798.

**Discussion:**

Developing psychometric tests for an OSCE in medication administration provides an empirical indicator that can be used accurately in nursing professionals' work.

**Conclusion:**

The validity of the OSCE for assessing competence in pediatric medication administration was established, and the instrument was deemed moderately acceptable for application in this area of knowledge. Its use favors both learning and evaluative processes of clinical practice for health sciences students.

## Resumen

**Introducción:**

La evaluación sistemática de las competencias clínicas del profesional de enfermería ha sido objeto de interés entre educadores, profesionales y otros expertos de esta área, lo cual posibilita rastrear el perfil de egreso, trayectoria y el alcance de las metas durante la carrera profesional. Los profesores deben priorizar la eficiencia en el desempeño de los futuros profesionales de enfermería mediante instrumentos como la simulación clínica para el desarrollo de la praxis.

**Objetivo:**

Determinar la validez de constructo del examen clínico objetivo estructurado para valorar la competencia de administración de medicamentos en pediatría.

**Materiales y métodos:**

Estudio observacional, psicométrico y prospectivo, en el que se adelantaron pruebas para determinar la validez y confiabilidad del instrumento: “Examen Clínico Objetivo Estructurado para la administración de medicamentos en pediatría”.

**Resultados:**

El instrumento final quedó conformado por 15 reactivos. La prueba de esfericidad de Bartlett´s fue significativa χ2=145,887 p <0,001 y el indicador de adecuación del tamaño de muestra Kaiser-Meyer-Olkin fue adecuado (0,703). El alfa del instrumento total fue de 0,798.

**Discusión:**

Desarrollar pruebas psicométricas al examen clínico estructurado para la administración de medicamentos, permite obtener un indicador empírico que puede ser utilizado de manera precisa en la labor del profesional de enfermería.

**Conclusiones:**

Se determinó la validez del examen clínico objetivo estructurado para valorar la competencia administración de medicamentos en pediatría, es un instrumento moderadamente aceptable para aplicar en esta área de conocimiento, favoreciendo el aprendizaje y los procesos evaluativos de la práctica clínica de los estudiantes de ciencias de la salud.

## Resumo

**Introdução:**

A avaliação sistemática das competências clínicas de profissionais de enfermagem tem sido fonte de interesse entre educadores, profissionais e outros especialistas da área. Isso permite o acompanhamento do perfil de graduação, da trajetória e do alcance de metas ao longo da carreira profissional. Os educadores devem priorizar a eficiência do desempenho dos futuros profissionais de enfermagem por meio de ferramentas como a simulação clínica para o desenvolvimento da prática.

**Objetivo:**

Determinar a validade de construto do exame clínico objetivo estruturado para avaliar a competência em administração de medicamentos em pediatria.

**Materiais e métodos:**

Foi realizado um estudo observacional, psicométrico e prospectivo para determinar a validade e a confiabilidade do instrumento "Exame Clínico Objetivo Estruturado para Administração de Medicamentos em Pediatria".

**Resultados:**

O instrumento final foi composto por 15 itens. O teste de esfericidade de Bartlett foi significativo (α2 = 145,887 p < 0,001) e o indicador de adequação do tamanho amostral de Kaiser-Meyer-Olkin foi adequado (0,703). O alfa do instrumento total foi de 0,798.

**Discussão:**

O desenvolvimento de testes psicométricos para o exame clínico estruturado para administração de medicamentos permite a obtenção de um indicador empírico que pode ser utilizado com precisão na atuação de profissionais de enfermagem.

**Conclusões:**

Foi determinada a validade do exame clínico objetivo estruturado para avaliar a competência em administração de medicamentos em pediatria. Este instrumento apresenta aceitação moderada para aplicação nesta área do conhecimento, favorecendo a aprendizagem e os processos avaliativos da prática clínica de estudantes de ciências da saúde.

## Introduction

Clinical simulation is an effective strategy for acquiring competencies in patient care^1^. The evaluation of clinical skills in future professionals has been a subject of study among health educators, as it enables the recognition of the profile, trajectory, and scope of the professional major^2^. Competency tests are results-oriented and evaluate individual performance based on professional knowledge and clinical skills. However, studies that assess clinical performance in nursing students are scarce^3^,^4^. Likewise, there is little reliability and validity in the assessment of clinical rotations, which suggests evaluation processes with subjective judgments, detached from reality^5^,^6^.

Professional nurses are distinguished by their importance and responsibility associated with the technique of care. In the administration of medication, a broad range of knowledge and skills must be developed, which are concrete abilities for professional practice^2^. Nurses are responsible for the preparation and administration of medications, a task considered one of the most delicate activities, given that errors are more frequent in hospitalized children and can result in patients' death^7^,^8^.

Educators must prioritize efficiency in evaluation processes in higher education by using instruments that specifically assess knowledge and practical skills^9^. Thus, it is necessary to have a tool that identifies existing competencies and what is expected to be achieved to ensure the safe and humane professional practice for patients^9^.

The evaluation of clinical skills is an essential tool for identifying professionals' profiles and determining if they demonstrate greater proficiency in cognitive, procedural, and attitudinal domains of knowledge^10^. Currently, clinical simulation laboratories are equipped to support the learning of future nurses, making it essential for faculty to reinforce clinical learning in these settings so that students acquire the necessary clinical knowledge and skills before entering the job market^11^,^12^.

One of the problems is that practice-based assessments are sometimes not fit for purpose; there are inconsistencies in interpreting the performance levels of nursing students and difficulty in providing constructive feedback during formative learning^5^,^6^. In this context, simulation enables the practice of medication administration in a clinical environment that resembles real life, while eliminating risks to patients. There, instructors guide learning, and acquired competencies are transferred into professional practice^13^-^15^.

In 1975, Harden introduced the Objective Structured Clinical Examination (OSCE) for assessing clinical competencies through direct observation across multiple structured stations using an evaluation checklist^16^. With this instrument, students demonstrate their abilities in a simulated, studied, and delimited clinical situation that takes place in a real clinical environment^3^,^4^. Several authors recognize the OSCE as an interesting alternative for evaluating the clinical performance of health science students. However, it faces significant challenges in ensuring the validity and reliability of the obtained results. Its main characteristic is adaptability across different contexts, along with the validation of its psychometric properties—indispensable requirements to determine whether the exam is summative; otherwise, its usefulness and precision are not guaranteed^17^. It has been used as a clinical assessment tool in undergraduate nursing education and rarely in postgraduate education^18^,^19^.

Therefore, the guiding question of this research was: What are the psychometric properties of an OSCE designed to evaluate the clinical competencies of nursing students in the "Child Health Care" course? The objective was to determine the validity and reliability of this tool for assessing competence in pediatric medication administration.

## Materials and Methods

**Design**

This was an observational, psychometric, and prospective study in which tests were conducted to determine the validity and reliability of the instrument: "Objective Structured Clinical Examination for pediatric medication administration." In this observational design, a checklist was used to record the presence or absence of behaviors and actions, grading each procedure as correct or incorrect. Students' scores were entered into a database. The questionnaire consisted of 20 items, each valued at 0.25 points for a maximum score of 5.0.

**Population and Sample**

For construct validation through exploratory factor analysis, the sample size was calculated following the recommendations of Campo and Oviedo^20^, who suggest including between five and twenty participants per item for twenty-item scales; that is, between 100 and 400 subjects. A total of 106 students were included in the study.

The inclusion criteria were being of legal age and being enrolled in the "Child Health Care" course, as the OSCE competencies were directly aligned with this course. Repeating the course was established as the sole exclusion criterion.

For expert validation, the inclusion criteria were a minimum of five years of experience in teaching and nursing care of children, particularly in the practice and instruction of pediatric medication administration. Based on these criteria, an email invitation was sent to fifteen nursing professionals, with seven of them fully completing the evaluation form.

**Variables**

The variables analyzed included sociodemographic variables (age and gender) and the variables of the OSCE, which were categorized by competencies. Cognitive competency included logical- mathematical thinking, mechanism of action, dose calculation, and infusion rate calculation. For praxiological competency, the identification and correct management of patient information were considered, including the appropriate administration of medication according to its type, dose, schedule, and route. Additionally, labeling, waste disposal, and documentation of medications were included. Communicative and attitudinal competency included confidence, empathy, autonomy, education, leadership, and teamwork.

**Procedure for Instrument Design**

The initial design of the OSCE comprised 25 items based on literature analysis as well as the teaching and clinical experience of the researchers. It included a student sheet with instructions and a clinical case to solve, and an evaluator's form with a binary checklist (correct and incorrect), covering the main actions to evaluate the clinical skill named“pediatric medication administration,” which includes theoretical and practical aspects.

The OSCE stations were developed according to the following stages:

**First stage:** Selection of the competency to be evaluated, using the syllabus of the "Child Health Care" course as a reference.

**Second stage:** Determination of the number of stations to be implemented according to the clinical skill to be developed.

**Third stage:** Development of support material for each station, consisting of multiple-choice questions with a single correct answer and the design of a clinical case. Additionally, the checklist was created.

**Fourth stage:** Selection of the most appropriate pediatric simulator, as well as the caregivers who prepared and rehearsed with the necessary supplies for pediatric medication administration.

The OSCE instrument was divided into three stations, each with its respective competencies. The first station assessed cognitive competencies and was divided into four dimensions: logical-mathematical thinking, mechanism of action, infusion rate calculation, and dose calculation. The second and third stations assessed praxiological, communicative, and attitudinal competencies, consisting of two clinical cases to be solved, one for each student (see supplementary material).

**Procedure for Instrument Application and Data Collection**

The students were enrolled in the "Child Health Care" course and had theoretical knowledge of pediatric medication administration. They attended three-day simulation laboratory sessions and were also given the option of voluntary unsupervised open practice sessions. They received access to the checklist, which detailed each skill and evaluation criteria.

Simultaneous scenarios were organized to ensure all details were obtained, providing reliable information based on established criteria. All assessments were conducted in the simulation laboratory of the Faculty of Health Sciences at the Universidad de los Llanos.

Students were prohibited from bringing any electronic devices to the examination. They were required to comply with laboratory regulations and could only enter with a lab coat, a stethoscope, a pencil, an eraser, and a pen. On average, each student completed three tests in approximately 60 minutes. This time included listening to the students' observations about the exam and their self-assessment.

At the second and third stations, students worked in pairs, supervised by an instructor, within a simulation scenario equipped with all necessary materials for the procedure. The scenarios included preparation and administration of intravenous and oral medications and blood products, as well as dose calculation based on the child's weight. Students were informed that the scenario represented a pediatric ward in a healthcare institution, and that they were to act in the role of a nurse. Clinical cases were randomly assigned to each pair, and students were evaluated independently of one another.

To assess communicative and attitudinal competencies, items were established to determine the student's confidence during the medication administration process, empathy and commitment in the act of caring, autonomy, and a critical and assertive attitude. Personal presentation, including proper uniform and a lab coat in perfect condition, was also assessed as part of the attitudinal competency.

Regarding communication, evaluators observed whether the student greeted the child and caregiver upon entering the unit, explained the purpose of their visit, and maintained a kind demeanor. They also assessed whether the student provided guidance and education regarding the pharmacological treatment, explaining to the parents or caregivers in clear, precise, and appropriate language the prescription and possible side effects or reactions of the medication being administered to the child, and whether they resolved doubts or made assertive decisions in each case.

Ten faculty members from the department participated as evaluators, completing the checklist by marking each procedure as correct or incorrect. Students' responses were documented in a database for subsequent analysis.

**Data Analysis**

After expert validation, the data were entered into an Excel database and analyzed using descriptive statistics to calculate the mean response score across all rated items and to estimate the content validity index and content validity ratio.

To estimate the construct validity of the OSCE, exploratory factor analyses (EFA) were performed. The Kaiser-Meyer-Olkin (KMO) measure of sampling adequacy and Bartlett's test of sphericity were calculated to confirm the presence of correlation patterns among the scale's items. The principal axis factoring extraction method with oblique rotation was used. Eigenvalues greater than 1 and factor loadings greater than 0.4 were considered significant and retained in the factor analysis^21^. The number of factors was selected following Kaiser's criterion of an eigenvalue greater than 1.

Internal consistency was evaluated using Cronbach's alpha, which assessed item-total correlations, squared multiple correlation (explained variance) with the scale's items, and the reliability coefficient if the item was deleted. These results are presented in Table 2. The overall instrument showed a reliability of 0.798, which is considered good. The complete dataset is available in Mendeley Data for open access and consultation^22^.

**Ethical Considerations**

The study complies with the guidelines outlined in Resolution 8430 of 1993 of the Colombian Ministry of Health for research involving human subjects. The study protocol was approved by the Bioethics Committee of the Universidad de los Llanos (record number 007, September 16, 2020).

## Results

**Content Validity of the OSCE**

Content validation was conducted with seven experts who agreed to participate in the process; six were women, and one was a man. The predominant undergraduate degree was a Bachelor of Science in Nursing, with one professional holding a Bachelor of Science in Pharmaceutical Chemistry. Regarding postgraduate training, four held Master's degrees in areas such as Pharmacology, Primary Health Care Research, and Nursing Research. The remaining experts were specialists in Pediatric Critical Care, University Teaching, and Internal Auditing of Health Institutions.

**Figure 1:**
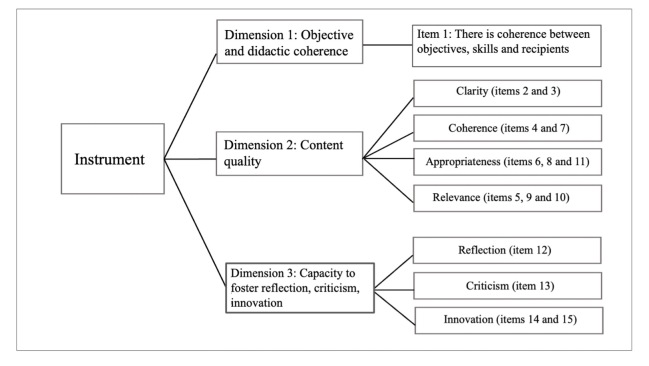
General structure of the instrument for content evaluation

The instrument used for expert judgment comprised three aspects or dimensions: objective and didactic coherence, content quality, and capacity to foster reflection. The second aspect involved revising the instrument's form to enhance clarity, coherence, pertinence, and relevance. Figure 1 presents the general structure of the instrument: dimensions and items presented to the expert judges. It should also be noted that items were rated on a 5-point Likert scale: 1 = disagree; 2 = partially disagree; 3 = partially agree; 4 = agree; and 5 = totally agree.

**Figure 2:**
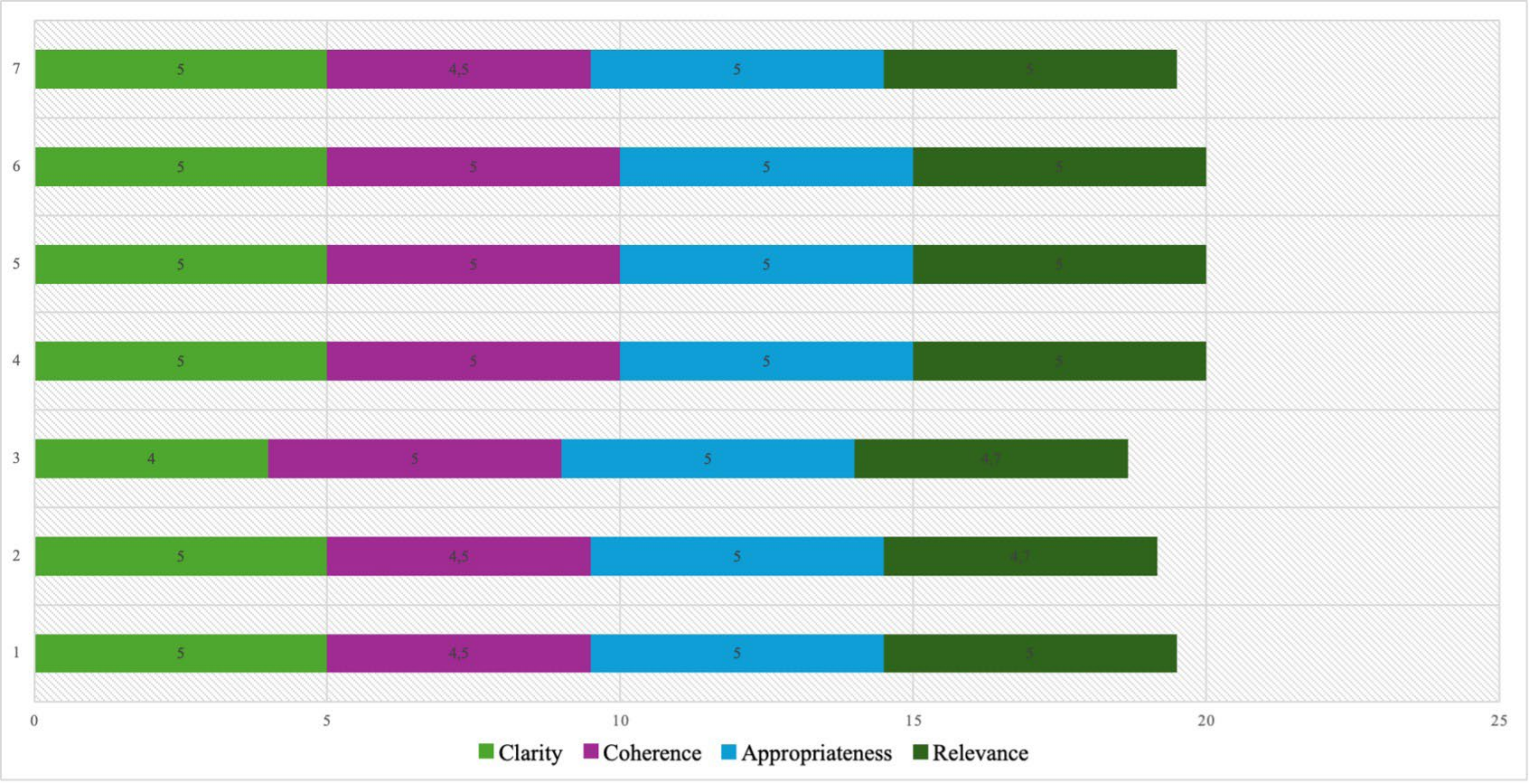
Evaluation of the aspects of dimension 2: Content quality

The first dimension was evaluated by all seven experts, with a maximum score of 5, indicating complete agreement regarding the coherence of the instrument's objective and didactics. Figure 2 shows the results for the content quality dimension. All four aspects of this dimension received a highly favorable average rating, interpreted as "totally agree," except for the clarity aspect, which was rated as "agree" by the third expert. Likewise, three experts (4, 5, and 6) assigned the maximum rating to all items of the evaluated aspects.

**Figure 3:**
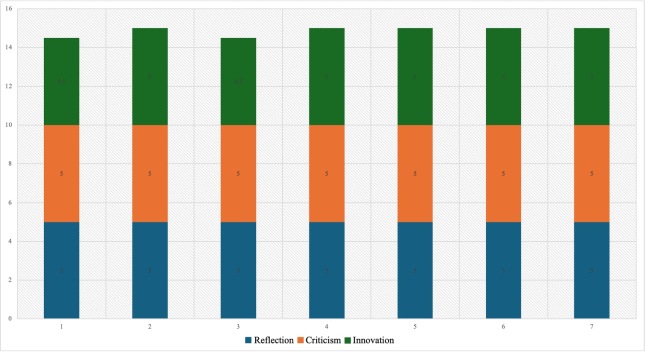
Evaluation of the aspects of dimension 3: Capacity to foster reflection, criticism, and innovation.

A similar situation was observed in the dimension “capacity to foster reflection, criticism, and innovation.” Figure 3 shows that the first and third experts assigned an average rating of 4.5 to the innovation aspect, which represented the lowest rating in only two items. The interpretation of ratings for this dimension was "totally agree" by all experts.

As general observations on the instrument, the experts mentioned that the design is practical for students to become familiar with the clinical scenario. The OSCE helps strengthen the training process of health professionals and materializes the flexibility required in higher education through the use of practical simulation-based tools.

Based on these results, the Content Validity Index (CVI) was calculated, ranging from 0.91 to 1.00. The fourth item, part of the coherence aspect of the content quality dimension, had the lowest score (0.91), followed by item 15 of the innovation aspect in the third dimension (0.94). Items 2 and 3 of the clarity aspect, as well as items 5, 9, and 10 of relevance, each obtained a CVI of 0.97. All items in the pertinence aspect (items 6, 8, and 11) reached a CVI of 1.0.

Regarding the CVIs of the three dimensions, the first dimension (objective and didactic coherence) had a CVI of 1.00; the second dimension (content quality) achieved a CVI of 0.97; and the last dimension (capacity to foster reflection, criticism, and innovation) had a CVI of 0.99. The overall CVI was 0.98. Likewise, a content validity ratio (CVR) of 1.00 was obtained for every item in the instrument.

In conclusion, the OSCE, with the adjustments made according to the observations of the seven experts, demonstrated excellent content validity, with CVIs above 0.90 and approaching 1.00. Nevertheless, the reliability of the information to be obtained through the evaluations will ultimately depend on the correct application of the instrument.

**Construct Validity of the OSCE**

Data were collected between April 2021 and September 2022. A total of 106 fifth-semester nursing students enrolled in the "Child Health Care" course participated. Of these, 73.58% (n=78) were women, and the mean age was 20.47 years (SD = 1.50).

**Figure 4:**
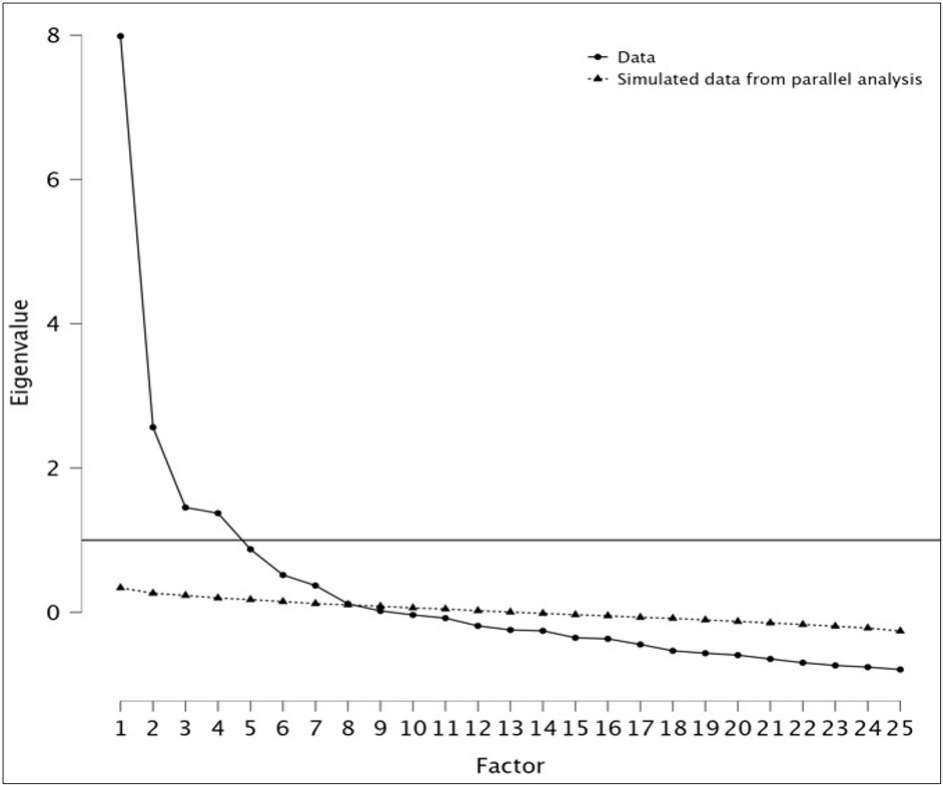
Scree plot of factor analysis

The validation of assumptions indicated that conducting an exploratory factor analysis was suitable. The Kaiser-Meyer-Olkin (KMO) measure of sampling adequacy presented a value of 0.703, and Bartlett's test of sphericity was significant (χ2 = 145.887, p < 0.001). According to Kaiser's criterion, seven factors were identified, explaining 72.90% of the variance. The result of this analysis prompted a second procedure. As shown in Figure 4, the first four factors accounted for most of the total variability of the data, as indicated by the eigenvalues.

The eigenvalues of the first five factors are greater than 1. The remaining factors explain only a small proportion of the variance and were deemed less relevant. The four-factor solution explained 72.30% of the common variance. The first factor included items reflecting safe actions prior to medication administration, corresponding to part of the praxiological dimension. The second factor, termed the communicative-attitudinal dimension, consisted of items with loadings ranging from 0.518 to 0.994. The third factor encompassed the items from the cognitive dimension, with factor loadings ranging from 0.417 to 0.935. Items 4, 5, 6, 7, 8, and 15 loaded onto factors different from the proposed theoretical construct and were therefore eliminated. The goodness-of-fit test showed a good model fit (χ2 = 66773.945, p < 0.001).

**Table 1:** Factor loadings using oblimin rotation for the items comprising the final instrument

Item	Factors			
	Factor 1 Praxiological 1^a^	Factor 2 Communicative-attitudinal	Factor 3 Cognitive	Factor 4 Praxiological 2^b^
V1			0.861	
V2			0.935	
V3		0.458	**0.483**	
V9			0.421	
V10	0.972			
V11	0.956			
V12	0.930			
V13			0.428	
V16			0.417	**0.665**
V17				0.794
V18				0.548
V19		0.684		
V20		0.729		
V21		0.994		
V22		0.518		

^a^ Corresponds to safe actions prior to medication administration ^b^ Corresponds to safe actions during and after medication administration

The results showed that the first factor grouped four of the 15 proposed items, corresponding to the praxiological dimension. The second factor grouped five items: one from the cognitive dimension, and four from the communicative-attitudinal dimension. The third factor integrated six items, five of which corresponded to the cognitive dimension. Finally, the fourth factor grouped the three items related to safe actions during and after medication administration from the praxiological dimension. Overall, there appears to be a preeminence of certain items for each dimension. However, despite this trend, the eliminated items spanned various dimensions and were dispersed across different factors.

Table 2 presents the reliability of the instrument, estimated using Cronbach's alpha reliability analysis for each dimension. The praxiological dimension corresponding to safe actions prior to medication administration shows a superior value. In contrast, the dimension referring to safe practice during and after administration presented the lowest value, indicating low internal consistency. A similar situation was observed with the communicative-attitudinal dimension, although its consistency is higher. The cognitive dimension showed acceptable consistency. The total instrument reported a high internal consistency of 0.798.

**Table 2:** Reliability of the final OSCE instrument

Factor	Dimension	Cronbach’s α	(95% CI) Lower limit	(95% CI) Upper limit
Factor 1	Praxiological 1a	0.928	0.919	0.935
Factor 2	Communicative-attitudinal	0.665	0.632	0.696
Factor 3	Cognitive	0.745	0.719	0.769
Factor 4	Praxiological 2b	0.627	0.585	0.666
**Total**	**OSCE**	**0.798**	**0.780**	**0.815**

^a^ Corresponds to safe actions prior to medication administration ^b^ Corresponds to safe actions during and after medication administration

## Discussion

Nursing professionals are committed to acquiring the necessary knowledge for the safe administration of pediatric medications, as even a minor error of administration can lead to adverse events in patients, compromising patient safety and quality of care. Bekes et al.^23^ consider that clinical skills are complex; consequently, objectively reliable instruments are required, especially in the clinical setting, whether in a hospital or a primary care center.

According to Alarcón^13^, evaluation is a driver of the learning process, highlighting the importance of including methods that meet the criteria of validity, objectivity, and reliability. For this reason, some authors are concerned about determining the validity and internal consistency of the OSCE in health education,^24^.

In Brazil, an instrument designed for patient safety in pediatric medication administration was developed and validated, yielding an index of 0.938 and a Cronbach's alpha of 0.851^8^, a finding similar to that of the present study. Thus, developing psychometric tests for an instrument provides an empirical indicator that can be used to measure nursing professionals’ work accurately^25^.

Although the OSCE is considered an effective tool for assessing clinical competencies, it causes stress in students and is both expensive and time-consuming^5^. However, contrary to this, the current study found that the participating students were satisfied with the experience and highlighted the importance of learning from their own mistakes and gaining greater confidence before entering real practice. This aligns with the findings of García et al.^26^, who reported that students enjoyed the activity, which positively influenced their academic performance. Furthermore, they recommended its frequent use, especially for measuring clinical competencies^2^. The OSCE conducted in skills labs proved to enrich students' clinical learning experiences^12^.

Hamui et al.^15^ consider that the consistent and systematic use of OSCEs can have a positive impact on the professional education of students, as this approach demonstrates that evaluation is a significant part of the educational process.

The OSCE validated in this research serves as a basis for creating a rubric with accurate language for assessing pediatric medication administration competencies. As a consensus-based rating, it has the potential to overcome problems with language comprehension or identifying the approved competency level, thereby managing to overcome the reliability obstacles in clinical practice evaluations reported in the literature^6^,^25^.

According to Montgomery et al.^19^, there is no consensus on the duration or number of OSCE stations, as these depend on the exam’s purpose, educational objectives, and the clinical skills being assessed. Likewise, the benefits derived from self-efficacy as a result of learning—recognizing safety, confidence, and competence in the clinical skill—cannot be denied.

Espinoza Fernández^17^ indicates the need to develop instruments that meet the criteria of validity and reliability. One validation conducted on measurement instruments is content validation. This process systematically compares test items with the learning of the content taught. According to Escurra^18^, this analysis can essentially be carried out by a panel of competent and qualified experts who will give an opinion on the instrument’s components.

The OSCE for pediatric medication administration can be used to evaluate the achievement of learning outcomes and professional competencies based on scientific evidence. Such instruments can be adapted to different contexts and levels of nursing education, while also strengthening the confidence of nursing professionals in their clinical skills. The content may vary depending on the student's experience, the nature of the assessment, and the type of problem commonly encountered in clinical practice^25^-^27^. Organizing the validation of the instrument required time, logistics, the use of human resources, and the availability of scenarios. Nevertheless, it is expected that this resource will be used to enhance the quality of nursing training for students at the Universidad de los Llanos and strengthen the program's curricular model, thereby giving meaning to teaching-learning theories, particularly in the evaluation of clinical competencies.

This study has limitations. The small sample size limited the analysis to only exploratory factor analysis, and the sample of students was drawn from a single university, thereby restricting the generalizability of the results. For future studies, combining in-person OSCE with virtual simulations is suggested to reduce costs and time. It would also be interesting to measure students' anxiety levels before and after the exam to identify the most stressful moments, and then redesign the stations accordingly. Additionally, implementing mock exams prior to the evaluation could help familiarize students with the methodology.

## Conclusions

This study demonstrates the validity of the objective structured clinical examination (OSCE) for assessing competency in pediatric medication administration. The total Cronbach’s alpha of 0.798 indicates that this instrument is moderately acceptable for use in this area of knowledge, thereby favoring the learning and accurate evaluation of clinical competencies in health sciences students. The OSCE is a costly instrument that can cause stress in students and requires time for planning and execution, as it needs additional human and material resources compared to conventional simulation- based training. Nevertheless, analyzing these barriers enables the identification of opportunities for improvement in teaching practices, curriculum development, and patient safety. Likewise, it is interesting to analyze the integration of active, student-centered methodologies that strengthen clinical judgment and the management of emotions in stressful situations.

## Supplemental Material

Objective Structured Clinical Examination Instrument for Pediatric Medication Administration

**UNIVERSIDAD DE LOS LLANOS **

**NURSING PROGRAM**

**CHILD HEALTH CARE COURSE**

**OBJECTIVE STRUCTURED CLINICAL EXAMINATION (OSCE)**

**SAFE PEDIATRIC MEDICATION ADMINISTRATION**

Name:_______________________ Age:_______ Date:_____________ Score:_________No 1

**STATION 1: Cognitive Competency**

Duration: 8 minutes

Setting: Classroom

Below are four questions, each with two answer options. Select the correct one. You have two minutes to answer each question.

Logical-Mathematical Thinking Dimension

Edwin has a certain number of playing cards. If he shares them with one friend, there is one card left over. If he shares them with two friends, there is one card left over. If he shares them with three friends, there is one card left over. If he shares them with four friends, there are none left over. How many cards does Edwin have?

A.24

B.25

Medication Dose Calculation Dimension

A 3-year-old child weighing 11 kg is diagnosed with severe broncho-obstructive syndrome. The pediatrician prescribes magnesium sulfate (presentation: 20% ampoule) at 50mg/kg to be administered immediately. The dose for the nurse to prepare is:

A.2.75 ml

B.5.5 ml

Mechanism of Action Dimension

Carbamazepine is an anticonvulsant commonly used in pediatrics. Its mechanism of action is:

A.Decreasing pre- and post-synaptic excitability, inhibiting the release of neurotransmitters.

B.Preventing repetitive firing of sodium-dependent action potentials.

Infusion Rate Calculation Dimension

The nurse needs to administer 10 mg of tramadol, diluted in 20 ml of 0.9% normal saline, via an infusion pump over half an hour. The rate that must be programmed is:

A.30 ml/h

B.40 ml/h

**STATION 2: Praxiological, Communicative, and Attitudinal Competencies**

Duration: 20 minutes

Setting: Simulation Laboratory

Below are two clinical cases. Read carefully the one indicated by the instructor, solve it, and then use the elements available in the laboratory. When finished, proceed to the patient's unit and administer the medication as if you were in a real scenario.

Be aware that you are on the pediatric ward of a healthcare institution with a capacity of 30 beds. There are two nurses on duty during the morning shift: one is responsible for half of the patients located in the north corridor, and the other for the remaining patients in the south corridor.

**Clinical Case 1 **(You have a total of 20 minutes)

Name: María Valentina Pérez Unit: 303 A Age: 15 months

Weight: 12 Kg Diagnosis: Right Basal Pneumonia

Medical Prescription: Ampicillin sulbactam 150mg/kg/day divided into four doses. Infuse for over 30 minutes.

**Clinical Case 2 **(You have a total of 20 minutes)

Name: Jesús Alberto Parra Unit: 3013 B Age: 12 months

Weight: 10 Kg Diagnosis: Seizure Disorder

Medical Prescription: Diazepam 3 mg IV once daily. Infuse for over 30 minutes. Schedule: 8 am

**UNIVERSIDAD DE LOS LLANOS CHILD HEALTH CARE COURSE**

**OBJECTIVE STRUCTURED CLINICAL EXAMINATION (OSCE) PEDIATRIC MEDICATION ADMINISTRATION CHECKLIST**

Name:_____________________ Age:_______ Date:_____________ Score:________

The evaluator, through direct observation, must mark with an X whether the student answered the questions and performed the requested procedures correctly or incorrectly. The examiner is limited to observing and should not pressure the student or provide additional information. Each item is worth 0.25 for a maximum score of 5.0.

**STATION 1 Cognitive Competency:**

Item Assessed	Correct	Incorrect
Question 1		
Question 2		
Question 3		
Question 4		

**STATION 2: Praxiological Competency:**

Item	Correct	Incorrect
5. Obtains information about the child's health condition for medication administration based on the shift report (e.g., patency of venous access) and clinical records.		
6. Administers the correct medication. Applies aseptic technique and biosafety standards throughout the procedure.		
7. Administers the correct dose. Knows how to calculate volume and infusion rate.		
8. Correct patient. (Before administering the medication, checks the child's name and identification.)		
9. Correct time. (Administers the medication at the established time; the limit is five to ten minutes before or after the set time).		
10. Administers via the correct route. (Ensures the administration route is the one indicated).		
11. Accurately labels the medication to be administered (unit number, child's name, medication, dose, date, time, and name of the person who prepared it) and places it in a visible location.		
12. Disposes of waste in the corresponding containers. (Places used needles and syringes in the sharps container, avoids risky acts such as attempting to recap used needles, places used cotton swabs or any other material contaminated with biological waste in the indicated container for that purpose, and does the same for non-contaminated materials).		
13. Correctly documents the administered medication. (Does so after administering the medication; it must be clear and contain the date, month, year, time, name of the medication, presentation, concentration, dose, frequency, route, and signature of the person who administered it).		

**STATION 3: Communicative and Attitudinal Competency:**

Procedure	Correct	Incorrect
14. Demonstrates confidence during the medication administration process.		
15. Demonstrates empathy and commitment in the act of caring (human, spiritual, and transpersonal behavior that the nursing student reflects in the practice of medication administration).		
16. Strengthens autonomy and demonstrates a critical and assertive attitude.		
17. Completes the OSCE with excellent personal presentation, the indicated uniform, and within the established time.		
18. Upon entering the unit, greets the child and caregiver, explaining the reason for their presence and maintaining a kind demeanor.		
19. Provides guidance and education on the pharmacological treatment. Explains to the parents or caregiver in clear, precise, and appropriate language the indication for which the medication will be administered to the child and the possible side effects or reactions resulting from its administration. Facilitates the caregiver's participation and resolves any doubts that may arise.		
20. Fosters and strengthens leadership and teamwork. (Defends the interests of the patients, oneself, and colleagues; knows how to communicate effectively and conveys confidence).		
